# Cortical Measures of Binaural Processing Predict Spatial Release from Masking Performance

**DOI:** 10.3389/fnhum.2017.00124

**Published:** 2017-03-21

**Authors:** Melissa A. Papesh, Robert L. Folmer, Frederick J. Gallun

**Affiliations:** ^1^Department of Veterans Affairs, National Center for Rehabilitative Auditory Research, VA Portland Healthcare SystemPortland, OR, USA; ^2^Department of Otolaryngology Head and Neck Surgery, Oregon Health and Science UniversityPortland, OR, USA

**Keywords:** human, hearing, electrophysiology, aging, evoked potential, binaural, speech

## Abstract

Binaural sensitivity is an important contributor to the ability to understand speech in adverse acoustical environments such as restaurants and other social gatherings. The ability to accurately report on binaural percepts is not commonly measured, however, as extensive training is required before reliable measures can be obtained. Here, we investigated the use of auditory evoked potentials (AEPs) as a rapid physiological indicator of detection of interaural phase differences (IPDs) by assessing cortical responses to 180° IPDs embedded in amplitude-modulated carrier tones. We predicted that decrements in encoding of IPDs would be evident in middle age, with further declines found with advancing age and hearing loss. Thus, participants in experiment #1 were young to middle-aged adults with relatively good hearing thresholds while participants in experiment #2 were older individuals with typical age-related hearing loss. Results revealed that while many of the participants in experiment #1 could encode IPDs in stimuli up to 1,000 Hz, few of the participants in experiment #2 had discernable responses to stimuli above 750 Hz. These results are consistent with previous studies that have found that aging and hearing loss impose frequency limits on the ability to encode interaural phase information present in the fine structure of auditory stimuli. We further hypothesized that AEP measures of binaural sensitivity would be predictive of participants' ability to benefit from spatial separation between sound sources, a phenomenon known as spatial release from masking (SRM) which depends upon binaural cues. Results indicate that not only were objective IPD measures well correlated with and predictive of behavioral SRM measures in both experiments, but that they provided much stronger predictive value than age or hearing loss. Overall, the present work shows that objective measures of the encoding of interaural phase information can be readily obtained using commonly available AEP equipment, allowing accurate determination of the degree to which binaural sensitivity has been reduced in individual listeners due to aging and/or hearing loss. In fact, objective AEP measures of interaural phase encoding are actually better predictors of SRM in speech-in-speech conditions than are age, hearing loss, or the combination of age and hearing loss.

## Introduction

Binaural hearing is a key contributor to improving speech recognition in background noise. The spatial information provided by binaural cues is key to the ability to segregate spatially separated auditory streams, thus allowing listeners to achieve better speech recognition at poorer signal-to-noise ratios compared to conditions without spatial separation between sound sources (Levitt and Rabiner, 1967; Freyman et al., 2001). This phenomenon is known as spatial release from masking (SRM) (Carhart et al., 1969). One of the fundamental binaural cues which facilitates SRM is the difference in the timing of acoustic information reaching the two ears when a sound source is offset to the left or right of a listener. When the wavelength of sound is larger than the diameter of the head, the difference in the phase of the acoustic waves reaching the ears can be detected and utilized to help localize a sound source. Thus, the upper frequency limit at which most listeners can benefit from interaural phase differences (IPD) for ongoing tones is approximately 1,500 Hz, which corresponds to a wavelength of approximately 22 cm, the average diameter of the human head. At higher frequencies with shorter wavelengths, such ongoing IPD cues become ambiguous, although onset cues and envelope cues can still be extracted and used for binaural discriminations (Stecker and Gallun, 2012).

Sensitivity to ongoing IPD depends both upon precise phase locking at each ear as well as accurate comparison of temporal differences between the two ears. Deficits in either of these two domains may result from hearing loss, aging, or a combination of the two (Moore et al., 2012; Gallun et al., 2014; King et al., 2014). For example, multiple studies have demonstrated that sensorineural hearing loss results in reduced ability to detect temporal fine structure cues compared to listeners with normal hearing, and that poor detection of temporal fine structure is correlated with poorer speech understanding particularly in difficult listening environments (Buss et al., 2004; Hopkins and Moore, 2011). Behavioral studies of the effects of hearing loss on SRM indicate that hearing loss interferes with the listeners' ability to detect the cues necessary for sound stream segregation, including temporal fine structure, leading to significantly poorer performance in conditions involving spatial separation between target and masking talkers compared to listeners with normal hearing sensitivity (Shinn-Cunningham and Best, 2008; Best et al., 2012; Gallun et al., 2014). In the case of aging, many lines of evidence indicate that it is accompanied by a general slowing of neural activity and a reduction in the accuracy of neural timing (Walton et al., 1998; Konrad-Martin et al., 2012). Consequently, the encoding and use of temporal information is impaired both in tasks of monaural temporal acuity and binaural interaural timing detection (Strouse et al., 1998; Gallun et al., 2014) leading to poor encoding of the temporal components of speech (Tremblay et al., 2002; Vander Werff and Burns, 2011; Anderson et al., 2012) reduced binaural listening benefit (Dubno et al., 2008), and reduced SRM (Warren et al., 1978; Gallun et al., 2013). Recent evidence indicates that the ability to detect binaural timing information actually begins to decline starting in middle age (e.g., 40–60 years of age) (Ross et al., 2007a; Grose and Mamo, 2010; Ruggles et al., 2011; Gallun et al., 2013, 2014; King et al., 2014), which is also the age at which people often begin to notice greater difficulty listening in noisy settings. Difficulty listening in noisy environments is further compounded by hearing loss, even when audibility has been restored with hearing assistive devices (Festen and Plomp, 1990; Divenyi and Haupt, 1997; Martin and Jerger, 2005; Helfer and Freyman, 2008). Because aging and hearing loss degrade sensitivity to binaural cues and binaural hearing is important to speech intelligibility in adverse listening environments, it is reasonable to assume middle-aged and older listeners, with and without hearing loss, experience increased difficulty understanding speech-in-noise in part due to reduced binaural information. However, few studies have previously examined this relationship. While there is a long history of behavioral testing of binaural function, evidence suggests that a significant training period and numerous repetitions are often required before binaural function can be reliably ascertained (Stecker and Gallun, 2012). This, combined with the increasing awareness that binaural function may be particularly sensitive to changes in temporal sensitivity (Strelcyk and Dau, 2009; Hopkins and Moore, 2010; Glyde et al., 2011) suggests that an objective measure would be of great utility.

An auditory evoked fields (AEF) paradigm utilizing magnetoencephalography has demonstrated sensitivity to the encoding of interaural phase cues (Ross et al., 2007b). Amplitude-modulated tones between 500 and 1,500 Hz were presented binaurally to young normal-hearing listeners. Stimuli began in phase between the two ears before a 180° IPD was introduced at the lowest amplitude portion of the stimulus. AEF data from the majority of subjects revealed clear responses to the IPD at carrier frequencies up to 1,250 Hz which were generally in good agreement with participants' behavioral limits of interaural phase discrimination of 1,200 Hz. Ross and colleagues then extended these findings to examine binaural processing in adults between the ages of 20 and 78 years of age (Ross et al., 2007a). Their AEF results indicated that the frequency limit at which adults could reliably detect the IPD was inversely proportional to age, with young adults detecting phase changes in carrier frequencies up to 1,225 Hz and older adults being unable to detect phase changes in frequencies beyond 760 Hz. Middle aged adults (average age of 50.7 years) had an upper limit of IPD detection of 940 Hz in spite of good pure tone hearing thresholds. The authors concluded that these data provide physiological support for the behaviorally established view that binaural hearing sensitivity begins to decline in middle age even before the onset of hearing loss.

The possibility of noninvasive physiological testing of binaural hearing sensitivity is enticing for those studying the neurological causes underlying speech-in-noise deficits in aging listeners, as well as clinicians invested in the assessment and rehabilitation of auditory concerns in this population. In the present studies, we (a) demonstrate that neural encoding of interaural phase difference information can be rapidly assessed using auditory evoked potentials (AEP), the electrical analog of AEF evoked using auditory stimuli, (b) explore the impacts of aging and hearing loss on IPD sensitivity using an AEP paradigm, and (c) examine the relationship between physiological measures of IPD detection and functional benefit from spatial cues to improve speech recognition performance. Functional benefit was assessed using a speech-in-speech recognition task with two conditions: one in which the speech maskers were co-located with the target talker, and one in which the speech maskers were spatially separated in the horizontal plane from the target talker. For the measurement of IPD responses, carrier frequencies included 750, 1,000, and 1,250 Hz as these frequencies were shown to reveal the limits of IPD encoding across a wide age range. We predicted that in listeners with intact hearing, older individuals would display poorer IPD sensitivity evidenced by less robust AEP responses compared to younger listeners, particularly at higher carrier frequencies. This relationship was expected to be even more evident in older listeners and those with hearing loss. Because individuals with intact binaural processing can take advantage of interaural timing differences to significantly improve their performance in the spatially separated task (Stecker and Gallun, 2012), we also predicted that objective AEP measures of listeners' sensitivity to binaural temporal cues would predict performance on tests of speech-in-noise with spatially separated maskers.

## Materials and methods

### Experiment #1

**Figure 1:**
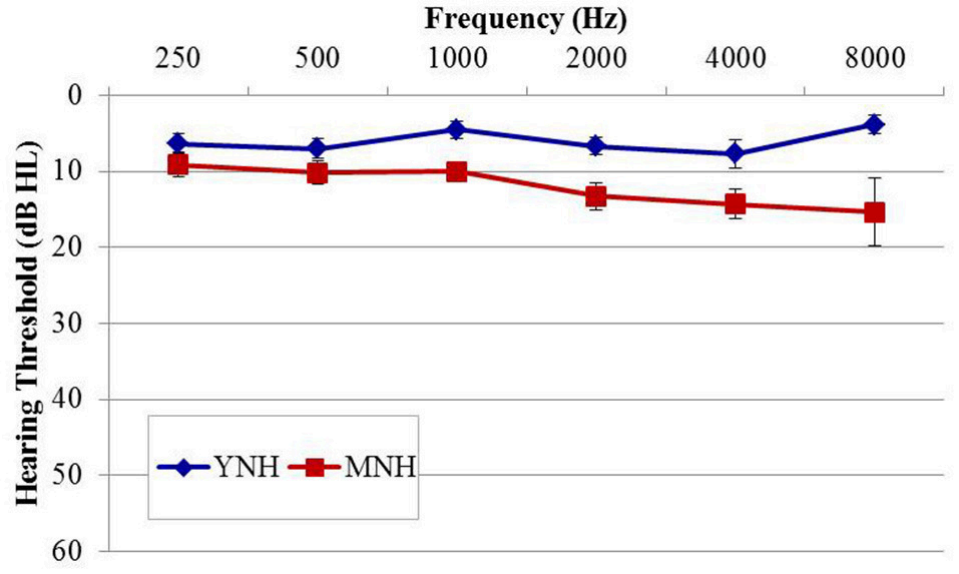
**Group mean audiograms averaged across ears for the young (YNH) and middle-age normal-hearing (MNH) participant groups in experiment #1**. Error bars indicate ± 1 SEM.

The purpose of experiment #1 was to probe sensitivity to IPD cues in listeners with essentially normal hearing thresholds using an AEP paradigm analogous to that used previously in AEF work by Ross et al. (2007b) Fifteen healthy younger normal-hearing (YNH) subjects (six female; mean age, 26.4 years; range of 22–32 years) and 14 healthy middle-aged normal-hearing (MNH) subjects (two female; mean age 51.1 years; range of 37–67 years) participated in the study. Participant eligibility criteria included native English speakers, intact mental status as defined by a score of 24 or better on the Mini Mental Status Exam (Tombaugh and McIntyre, 1992), no evidence of conductive or retrocochlear hearing loss, and no asymmetrical hearing exceeding 10 dB at more than one audiometric frequency below 4 kHz. Group averaged audiometric data are shown in Figure 1. In the YNH group, all participants had octave pure-tone thresholds between 250 and 4,000 Hz of 15 dB HL or better with the exception of one individual who had a 30 dB HL threshold in the left ear at 4,000 Hz. In the MNH group, all measured thresholds at octave frequencies between 250 and 4,000 Hz were 25 dB HL or better with the exception of one participant who had thresholds of 30 and 35 dB HL in the left and right ears, respectively. A one-way ANOVA based upon pure tone average (PTA) thresholds at octave frequencies between 500 and 8,000 Hz in both ears revealed that the MNH group had significantly poorer pure tone thresholds than did the YNH group by approximately 6.7 dB [*F*_(1, 27)_ = 13.295; *p* = 0.001]. Participant age was significantly correlated with PTA tone thresholds (*r* = 0.534; *p* = 0.003). All participants provided their informed consent prior to taking part in the study, which was approved by and conducted in accordance with the recommendations of the Department of Veterans Affairs Portland Healthcare System Institutional Review Board. All subjects gave written informed consent in accordance with the Declaration of Helsinki.

#### Auditory evoked potential stimuli and procedures

**Figure 2:**
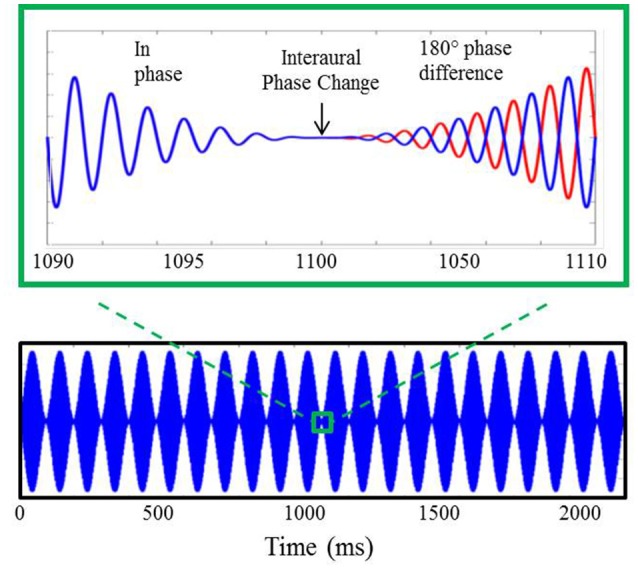
**Example of auditory stimuli used during AEP testing**. The bottom panel displays a 750 Hz carrier tone amplitude modulated at 10 Hz lasting for a duration of 2.2 s. A 20 ms section of the stimulus surrounding the 180° IPD is shown in the upper panel where, after the interaural phase change, the blue line represents the phase of the stimulus presented to the left ear and the red line indicates the phase of the stimulus presented to the right ear.

Stimuli for the electrophysiological component of experiment #1, shown in Figure 2, were based upon those utilized in previous AEF investigations described above (Ross et al., 2007a,b). They included sinusoidally amplitude-modulated (SAM) tones of 2.2 s in duration presented repeatedly with an interstimulus interval (from offset to onset) of 4.5 s. The SAM tones, presented binaurally to listeners through Etymotic ER3A insert earphones, were diotic for the first 1.1 s at which time the phase of the carrier frequency was shifted 180° in one ear relative to the other. This IPD occurred during a zero-amplitude instance of the amplitude modulation cycle. The sole purpose for amplitude modulating the carrier tones was to eliminate the potential for “spectral splatter” produced by the sudden initiation of the phase shift. Presenting the phase shift during a zero-amplitude instance in the modulation cycle ensured that neural response to the IPD stimuli were based solely upon the strength of phase locking to the fine structure of the carrier frequency and were not influenced by off-frequency cues or changes in stimulus envelope. All listeners heard stimuli 100% amplitude modulated at a rate of 10 Hz. For the sake of comparison with previous AEF work, a subset of each group also heard stimuli modulated at a rate of 40 Hz which was identical to the rate used by Ross and colleagues, though this data was not further analyzed beyond presentation of the grand averaged responses to this rate. A modulation rate of 10 Hz rather than 40 Hz was adopted in the present study as the slower rate allowed for more gradual ramps before and after the IPD shift at the zero-amplitude point, thereby reducing the possibility of listeners detecting a monaural cue. Nine participants in the YNH group (two female; mean age 26.9 years; range of 22–32 years) and nine participants in the MNH group (two female; mean age 54.7 years, range of 43–67 years) were presented with 40 Hz modulation stimuli in addition to 10 Hz modulation stimuli. Results were qualitatively similar to those obtained in the previous AEF studies and to those reported below, giving confidence in the use of a 10 Hz modulation rate.

Carrier frequencies of 750, 1,000, and 1,250 Hz were presented to both listener groups. These carrier frequencies were selected based upon AEF studies which demonstrated good correspondence to IPD detection thresholds in young, middle-aged, and older adults, respectively (Ross et al., 2007b). Stimuli were presented at a level of 85 dB sound pressure level (SPL) calibrated using a manikin with built-in ear simulators (Brüel and Kjær, Denmark). Each carrier frequency at a given modulation rate was presented a total of 100 times within an experimental block, with each experimental block lasting approximately 10 min. For participants who were tested using the 10 Hz modulation rate only, AEP data collection lasted approximately 30 min. Those who were tested using both 10 and 40 Hz modulation rates underwent AEP testing for approximately 1 h. During testing, participants were seated comfortably in a reclining arm chair positioned within a sound attenuating room. They watched a closed-captioned movie of their choice and were encouraged to take breaks as needed to maintain alertness.

AEPs were recorded from 15 Ag-AgCl scalp electrodes using a Quik Cap (Compumedics, Charlotte, NC). Scalp electrode positions were Fpz, Fz, Cz, Pz, Oz, F3, F4, C3, C4, P3, P4, T5, T6, T7, and T8 according to the International 10–20 System. The ground electrode was located on the scalp between electrode positions Fz and Ppz; the reference electrode was placed on the subject's nose. Horizontal and vertical eye movements were monitored with electrodes located above, below, and at the outer canthi of both eyes. Using a Neuroscan (Compumedics, Charlotte, NC) recording system, all channels were amplified and converted using an analog-to-digital sampling rate of 1,000 Hz. The recording time window consisted of a 100-ms prestimulus baseline followed by 3 s post-stimulus epoch. Evoked responses were analog band-pass filtered on-line from 0.15 to 100 Hz (12 dB/octave roll off). Continuous raw data files were further filtered offline from 0.1 Hz (high-pass filter, 24 dB/octave) to 30 Hz (low-pass filter, 12 dB/octave) using a zero-phase filter, and trials with eyeblink artifacts were corrected off-line using Neuroscan software. This blink reduction procedure calculates the amount of covariation between each evoked potential channel and a vertical eye channel using a spatial singular value decomposition and removes the vertical blink activity from each electrode on a point-by-point basis to the degree that the evoked potential and blink activity covaried (Neuroscan Inc., 2007). After blink correction, trials containing artifacts exceeding ±70 μV were rejected before the remaining epochs were averaged. For all individuals and conditions, 70% or more of the collected trials were available for averaging after artifact rejection.

The peak latency and amplitude values of waves N1 and P2 in response to the IPD were obtained from the central electrode Cz by an experienced judge relying on five methods. The primary method was the selection of peaks that were clearly larger than the response to the amplitude modulation. In conditions where this method did not produce an obvious peak, the following four methods were employed. Method 2: the response at electrode location Cz was compared with the responses at the temporal electrode sites. Because N1 and P2 are generated by auditory cortex, when responses at electrodes locations at and below the temporal lobe are assessed, they appear to be upside down, or inverted, compared to responses at vertex (Cz). This is because they are on the opposite side of the dipole that generates N1 and P2 peaks (superior temporal sulcus) from Cz. If an inverted peak is not found at the same time point on the temporal electrodes, this would indicate that the “peak” found at the vertex electrode is either noise, or from a source that does not have the same dipole signature as auditory cortex. This distinguishes the IPD or onset peak from noise, but not necessarily from responses to amplitude modulation. Method 3: responses from the carrier frequency condition in question were compared with responses to the other carrier frequency conditions to ensure that the peak in question was characteristic of the individual's N1 or P2 response. Method 4: global field power, which is based on the absolute value of the response waveform, was examined in order to confirm the latency of the peak in question. Finally, if questions still remained, Method 5 was used: responses for an individual were compared with those of the grand average for their group. If this set of five methods did not produce clear evidence of a peak, it was marked as absent. Amplitude values were measured relative to baseline and latency measures were determined relative to signal onset. To ensure that these methods of peak picking were followed, a random sample of 30% of the data was analyzed by two independent experimenters. Comparison of the resulting peaks revealed excellent correspondence between investigators such that only three IPD peaks were significantly different (quantified as more than 15 ms difference in latency) out of approximately 70 resampled peaks. These mismatches occurred when assessing responses to near-threshold presentation levels when one rater selected a peak while the other indicated an absent peak. To ensure that only the most reliable peaks were utilized, those peaks for which the raters disagreed on the presence or absence of a response were omitted from further analysis. Overall, the close correspondence between peaks selected by each independent rater suggests excellent adherence to the peak-picking methods described above.

#### Behavioral stimuli and procedure

Detection of IPD allows listeners to take advantage of spatial separation between an auditory signal of interest and conflicting sound sources. Thus, a goal of experiment #1 was to determine whether physiological detection of the IPD was predictive of a listener's ability to benefit from a spatial separation between a target talker and two masking talkers to improve speech recognition thresholds. All stimuli were presented over ER-2 insert earphones (Etymotic Research, Elk Grove Village, IL), and SRM was operationalized as the difference between a condition in which three talkers were all virtually presented from the same location (“co-located”) directly in front of the listener and a condition in which all three talkers were virtually presented from different locations (“spatially separated”). In the spatially separated condition, the target talker was virtually located directly in front of the listener while the two concurrent masking talkers were virtually presented at 45° angles to the right and left of the listener. The specific stimuli and methods used to probe SRM have been previously described in detail by Gallun et al. (2013, experiment 3). Briefly, stimuli consisted of sentences from the Coordinate Response Measure (CRM) corpus (Bolia et al., 2000) which follow the format “Ready (CALL SIGN) go to (COLOR) (NUMBER) now.” Listeners heard three simultaneously presented CRM sentences and were instructed to listen for the “Charlie” call sign and then press a button corresponding to the associated color and number. Both color and number needed to be correctly identified for a trial to be counted as correct. Eight different call signs were available (including “Charlie”) as well as four color options and the numbers one through eight. Three prerecorded male talkers spoke all target and masker sentences (Talkers 1, 2, and 3 of the CRM corpus). Spatial separation of sentences was achieved by using a virtual spatial array which employs head-related transfer functions (HRTF) to simulate spatial aspects of sound (Xie, 2013). The HRTFs were from the CIPIC database (http://interface.cipic.ucdavis.edu/sound/hrtf.html) and were measured with a binaural manikin. Custom MATLAB functions were used to convolve each sentence recording with the impulse response functions associated with the left and right ear HRTFs. The virtual source distance from the listener was 1 meter. In the co-located condition, both the target and the masker sentences were convolved with HRTFs recorded at the 0° azimuth in order to simulate sound directly in front of the listener. In the spatially separated condition, the target sentence was virtually presented at 0° azimuth while one masker was virtually presented at −45° and the other at +45°. The level of the target remained fixed at 39.5 dB above the speech recognition threshold for that listener (sensation level; SL) while the levels of the masker stimuli progressively increased across trials. Target-to-masker ratio (TMR) represents the relative level of the target to one of the maskers, and is more appropriate than the ratio of the target to the summed level of both maskers in tasks where confusions among speech signals limit performance rather than when performance is limited by the audibility of the target speech (Kidd et al., 2008; Marrone et al., 2008).

TMR thresholds were estimated by presenting 20 trials, two at each of ten target-to-masker ratios, starting at 10 dB TMR and dropping to −8 dB TMR in 2 dB steps. For both the co-located and the spatially separated conditions, the number of correct trials was then subtracted from ten to estimate the participant's TMR thresholds. This estimate has been found to closely approximate TMR obtained with a more extensive adaptive tracking procedure (Gallun et al., 2013), although thresholds are underestimated for participants near the edges of the range (beyond TMR values of +6 and −6 dB). Results shown below (**Figure 5**) indicate that these TMR thresholds were observed for no more than three or four of the participants across both experiments #1 and #2.

#### Analysis

For the purposes of statistical analysis, individual thresholds were averaged at octave frequencies between 500 and 8,000 Hz for both ears, yielding a pure tone average (PTA). Measures of PTA threshold, speech perception performance for both conditions and overall SRM, and latencies and amplitudes of IPD response components at each frequency for each group were submitted to one-way ANOVA. Only responses to the 10 Hz amplitude modulated AEP stimuli were analyzed, while the 40 Hz amplitude modulated stimuli served only as a comparison to previous AEF work conducted using SAM stimuli at the 40 Hz modulation rate. The strength and significance of the relationships between age and PTA threshold and the amplitudes and latencies of IPD responses at each test frequency were determined using bivariate correlations. To balance the exploratory nature of this analysis with the possible inflation of the chance of false discovery, the *p*-values are reported both with and without correction for multiple comparisons. Multiple linear regression was employed to determine the degree to which combinations of age, PTA thresholds, and IPD responses could predict performance on the spatially separated condition of the speech-in-noise recognition task and overall SRM. All statistical analyses were conducted using IBM SPSS Statistics 22 software package.

### Experiment #2

In experiment #2, sensitivity to binaural phase shift was probed in a group of 33 adults with a greater extent of hearing loss. It was hoped that by examining a group of listeners over 50 years old with a wider range of hearing thresholds it would be possible to more clearly discern the effects of hearing loss relative to the effects of age. All participants provided their informed consent prior to taking part in the study, which was approved by and conducted in accordance with the recommendations of the Department of Veterans Affairs Portland Healthcare System Institutional Review Board. All subjects gave written informed consent in accordance with the Declaration of Helsinki.

#### Materials and methods

**Figure 3:**
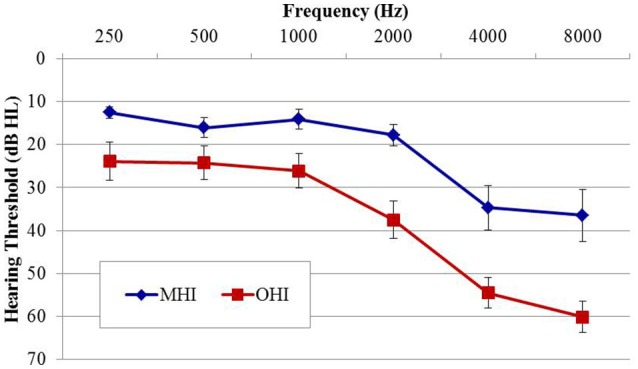
**Group mean audiograms averaged across ears for the middle-age (MHI) and older hearing-impaired (OHI) participant groups in experiment #2**. Error bars indicate ± 1 SEM.

Participants in experiment #2 were divided into two age groups to facilitate comparison of the effects of age on physiological IPD responses. The MHI group consisted of 17 individuals (four female; mean age 60.8 years; range 53–65 years) and the OHI group consisted of 16 individuals (one female; mean age 71.4 years; range 66–79 years). Participant eligibility criteria included native English speakers, intact mental status as defined by a score of 24 or better on the Mini Mental Status Exam (Tombaugh and McIntyre, 1992), absence of a current major medical disease (e.g., cancer, renal disease, end-stage cardiopulmonary disease, diabetes), no history of head trauma, neurological, or psychiatric disorders, and no current or recent history of drug or alcohol abuse. Group averaged audiometric data are shown in Figure 3. As in experiment #1, PTA was taken as the average of thresholds for both ears at octave frequencies between 500 and 8,000 Hz. A one-way ANOVA revealed significantly better PTA hearing thresholds in the MHI group compared to OHI group by an average of 16.7 dB [*F*_(1, 31)_ = 14.414; *p* = 0.001], and a significant correlation was found between age and average pure tone threshold (*r* = 0.560; *p* < 0.001). All participants provided their informed consent prior to taking part in the study, which was approved by the Department of Veterans Affairs Portland Healthcare System Institutional Review Board (IRB). Because experiment #2 was conducted separately from experiment #1 and required a separate informed consent document, IRB regulations prevent us from combining or directly comparing data from experiment #1 with data obtained in experiment #2.

#### Auditory evoked potential stimuli and procedures

All AEP stimuli and procedures in experiment #2 were identical to those in experiment #1 with the exception that only 10 Hz modulation rate stimuli were used.

#### Behavioral stimuli and procedure

All behavioral stimuli and procedures included in experiment #2 were identical to those described in experiment #1.

## Results

### Experiment #1

**Figure 4:**
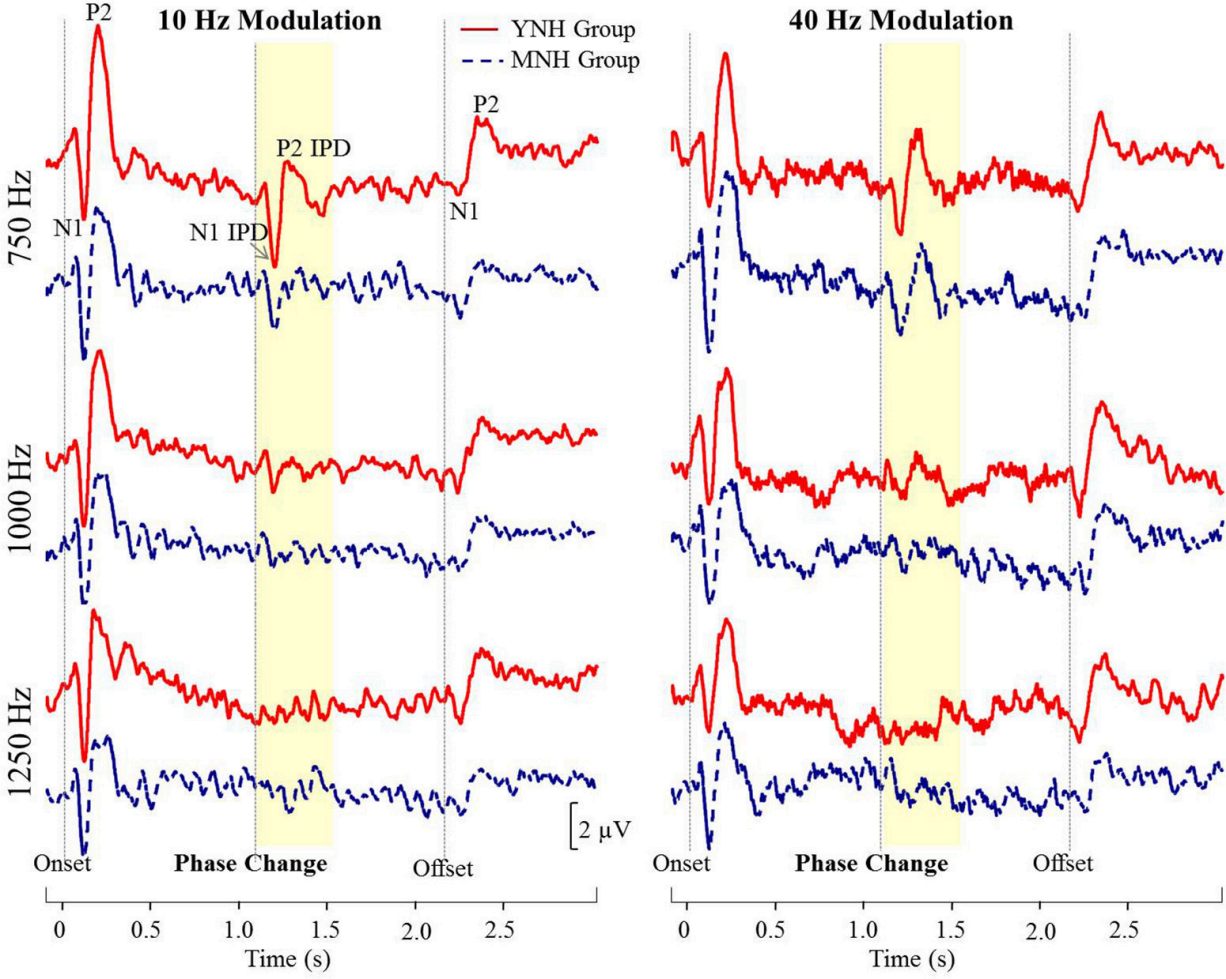
**Grand averaged waveforms for each group in experiment #1**. The yellow segment highlights the portion of the waveforms reflecting response to the interaural phase difference (IPD). For reference, N1 and P2 peaks in response to stimulus onset, IPD, and offset are labeled for the young normal-hearing (YNH) group in response to the 750 Hz carrier at 10 Hz modulation.

Figure 4 shows grand averaged waveforms in response to each of the three carrier frequencies modulated at both 10 and 40 Hz, with YNH participant averages shown in red and MNH participant averages shown in dashed blue. Both groups showed strong N1 and P2 onset and offset responses across all carrier frequencies and modulation rates. N1 and P2 waves in response to the IPD are clearly identifiable in the 750 Hz condition at both modulation rates for both subject groups. In the 1,000 Hz conditions, these IPD components are present though smaller in amplitude, and are absent in the 1,250 Hz conditions. Overall, the waveform morphology is similar between the 10 and 40 Hz modulation conditions. Thus, because responses to the 10 Hz modulation rate were obtained on a larger sample size and because of the reduced risk of monaural cues confounding results, all further analyses were conducted on the 10 Hz data.

N1 and P2 latencies and amplitudes were measured from the vertex electrode in response to stimulus onset, the IPD that occurred 1,100 ms post-stimulus onset, and stimulus offset. Group averaged means and standard deviations of these values are shown in Table 1 in response to the 750 and 1,000 Hz stimulus. The response latencies are presented relative to stimulus onset, but by subtracting 1,100 ms the IPD responses can be converted to latencies relative to the IPD and by subtracting 2,200 the offset responses can be converted to latencies relative to stimulus offset. These adjustments reveal that the average N1 peaks are close to 100 ms after the relevant stimulus event for onsets (116–120 ms), slightly longer for IPDs (130–160 ms), and shortest for offsets (103–117 ms). Similar patterns are seen for the P2 peaks. Individual responses to the 1,250 Hz stimulus were not scored because most study participants did not demonstrate identifiable responses to the IPD. All but one of the YNH participants had identifiable N1 and P2 peaks in response to the IPD at the 750 Hz carrier frequency, and all but two of the MNH participants had discernable IPD responses to this stimulus. Note that one MNH participant had a corrupt data file in response to the 750 Hz carrier modulated at 10 Hz. Thus, for this condition, the MNH participant group consisted of 13 total participants. For the 1,000 Hz carrier frequency, nine YNH participants had discernable IPD responses and six did not, while four MNH participants had discernable responses and ten did not. Though younger listeners were generally more likely to have discernable responses to the 1,000 Hz IPD stimulus than the middle-aged listeners, Chi-Square analysis failed to reveal a significant group difference (χ^2^ = 2.892, df = 1, *p* = 0.089). Younger participants also generally had larger N1 and P2 IPD amplitudes in response to the 750 Hz and 1,000 Hz stimuli compared to middle-aged participants. However, one-way ANOVA indicated that these effects failed to reach significance [N1 IPD at 750 Hz: *F*_(1, 23)_ = 4.165, *p* = 0.053; P2 IPD at 750 Hz: *F*_(1, 23)_ = 2.878, *p* = 0.103; N1 IPD at 1,000 Hz: *F*_(1, 11)_ = 1.164, *p* = 0.304; P2 IPD at 1,000 Hz: *F*_(1, 11)_ = 1.553, *p* = 0.239]. Peak IPD latencies were similar for both YNH and MNH listeners, and no significant group differences were found for either the 750 or 1000 Hz stimuli. No significant group differences were found between the latency or amplitude of N1 onset responses [*t*_(26)_ = −0.128, *p* = 0.899; *t*_(26)_ = −0.170, *p* = 0.867, respectively) nor P2 onset responses [*t*_(26)_ = −0.605, *p* = 0.551; *t*_(26)_ = −0.854, *p* = 0.401, respectively], thus bolstering the conclusion that group differences on IPD measures were in fact representative of IPD encoding ability and simply due to differences in general AEP responsivity among participants.

**Table 1:** **Group mean (SD) latency and amplitudes of N1 and P2 peaks in response to 750 and 1000 Hz stimuli amplitude modulated at 10 Hz in experiment #1**.

		**Onset**		**IPD**		**Offset**	
		**Latency (ms)**	**Amp (μV)**	**Latency (ms)**	**Amp (μV)**	**Latency (ms)**	**Amp (μV)**
**N1 PEAKS**							
750 Hz	YNH	116.4 (10.3)	−2.79 (2.0)	1,232.1 (25.4)	−4.6 (2.6)	2,303.5 (19.2)	−2.74 (2.0)
	MNH	116.9 (11.2)	−2.67 (1.7)	1,244.6 (42.2)	−2.73 (1.7)	2,303.0 (10.0)	−2.35 (1.4)
1000 Hz	YNH	116.9 (14.1)	−2.50 (1.6)	1,257.4 (37.3)	−3.15 (2.0)	2,317.3 (46.9)	−2.29 (2.5)
	MNH	120.1 (11.4)	−2.27 (1.3)	1,238.4 (35.5)	−1.96 (1.3)	2,310.4 (24.3)	−1.17 (1.0)
**P2 PEAKS**							
750 Hz	YNH	192.2 (28.3)	2.91 (1.3)	1,308.9 (25.5)	−0.4 (1.6)	2,404.7 (25.5)	1.26 (1.3)
	MNH	197.7 (17.6)	3.34 (1.4)	1,338.1 (58)	0.57 (1.0)	2,428.3 (34.1)	1.67 (0.89)
1000 Hz	YNH	182.2 (15.3)	2.14 (1.6)	1,340.8 (58.6)	−0.50 (1.9)	2,397.7 (29.1)	1.06 (1.2)
	MNH	197.0 (23.8)	3.38 (1.2)	1,326.1 (55.8)	0.76 (0.6)	2,412.9 (20.5)	1.69 (0.8)

*Subject groups included young normal-hearing (YNH) and middle-age normal-hearing (MNH) participants. Data are shown for responses to stimulus onset, interaural phase shift (IPD) occurring 1,100 ms post-stimulus onset, and stimulus offset occurring at 2,200 ms*.

**Figure 5:**
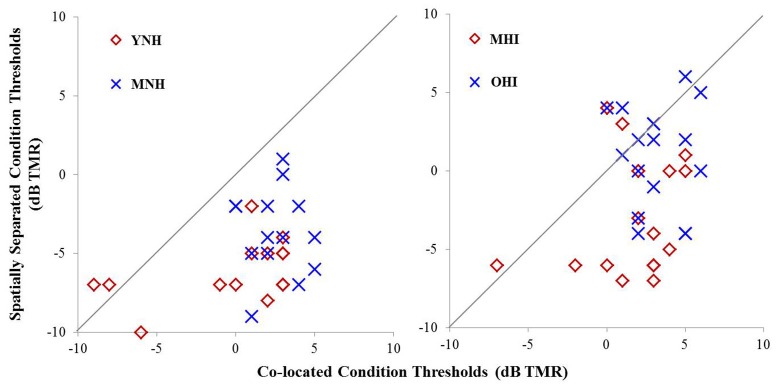
**Performance on speech understanding in noise task among participants in experiment #1 (left panel)** and experiment #2 **(right panel)**. Thresholds obtained in the co-located condition are plotted as a function of thresholds obtained in the spatially separated condition. The gray line indicates unity between thresholds obtained in the two conditions.

Individual TMR thresholds measured in both the co-located and the spatially separated conditions of the speech-in-noise task are plotted in Figure 5 (left panel). All but two individuals achieved better thresholds in the spatially separated condition compared to the co-located condition (thresholds below the line of unity). The two individuals who did not achieve better performance in the spatially separated condition were MNH participants who had excellent thresholds in both conditions, and thus had little room for improvement beyond the thresholds obtained in the co-located condition. Overall, the YNH listener group achieved an average TMR threshold of −0.2 dB (SD: 4.1 dB) in the co-located condition while the MNH participants performed slightly poorer with an average threshold of 2.5 dB (SD: 1.7 dB). In the spatially separated condition, the YNH participants achieved an average threshold of −6.1 dB (SD: 2 dB) while the MNH listeners had an average threshold of −3.6 dB (SD: 2.7). Group differences were significant for both co-located [*F*_(1, 27)_ = 5.242; *p* = 0.030] and spatially separated [*F*_(1, 27)_ = 7.849; *p* = 0.009] conditions. However, SRM, determined by subtracting an individual's threshold obtained in the spatially separated condition from their threshold obtained in the co-located condition, revealed a high degree of similarity between the groups with the YNH participants benefiting an average of 5.9 dB between the two conditions (SD: 3.8 dB) and the MNH group benefiting an average of 6.1 dB (SD: 3.3 dB). The lack of group differences in overall SRM likely reflect the few YNH participants who had TMR thresholds better than −5 dB in the co-located condition and thus were limited in amount of improvement they could achieve in the spatially separated condition.

**Figure 6:**
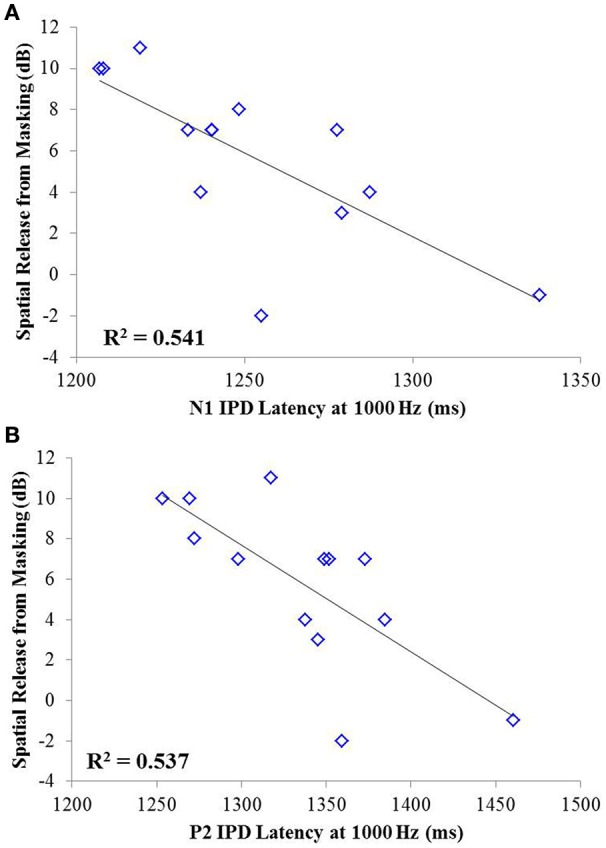
**Significant correlations found between IPD measures and SRM in experiment #1 at the *p* < 0.001 criterion level**. **(A)** Correlation between spatial release from masking (SRM) and N1 IPD latency in response to the 1000 Hz stimulus. **(B)** Correlation between SRM and the P2 IPD latency in response to the 1000 Hz stimulus.

Correlation analysis was conducted assessing the strength and significance of relationships between age, pure tone audiometric thresholds, speech recognition in noise, and the latencies and amplitudes of the N1 and P2 components of the IPD in response to 750 and 1,000 Hz stimuli. Correlation coefficients for these analyses are presented in Table 2, with the *p*-values indicated by the number of asterisks. Due to the unequal numbers of participants with scorable responses to the various stimuli, there is variability in the number of values included in the various correlations. Relationships found to be significant at the *p* < 0.05 criterion level are bolded with the respective *p*-values shown below in parentheses. Due to the exploratory nature of the present study, the *p*-values are provided with the recommendation that a value less than 0.05 is indicative of a potential relationship worthy of future investigation. Use of the highly stringent Bonferroni correction indicated a significance cut-off of *p* ≤ 0.001 (accounting for 51 comparisons). No correlations in experiment #1 were found to meet significance using the Bonferroni correction. However, between SRM and the latency of both N1 and P2 IPD peaks in response to 1,000 Hz stimuli (Figures 6A,B, respectively) were found to be significant at the *p* < 0.01 level. Additional positive correlations based on uncorrected *p*-values were found between participant age and TMR thresholds in the co-located and spatially separated conditions of the speech recognition task as well as between age and the latency of the P2 IPD response to the 750 Hz stimulus, and between pure tone audiometric thresholds and the latency of the N1 IPD response to the 750 Hz stimulus, and between SRM and the combined peak-to-peak amplitude of N1 and P2 IPD peaks in response to 750 Hz stimuli. No other correlations were found to be significant.

**Table 2:** **Correlations (*r*-values) between performance on each condition of the speech recognition task and age, pure tone average (PTA) hearing threshold, and measures of the interaural phase difference protocol for participants in experiment #1**.

	**Age**	**PTA**	**IPD Measures: 750 Hz**					**IPD Measures: 1000 Hz**				
			**N1 Lat**.	**P2 Lat**.	**N1 Amp**.	**P2 Amp**.	**N1/P2 Amp**.	**N1 Lat**.	**P2 Lat**.	**N1 Amp**.	**P2 Amp**.	**N1/P2 Amp**.
Age	–	**0.534 (*****p*** = **0.003)**	0.322	**0.523 (*****p*** = **0.007)**	0.352	0.312	−0.209	−0.271	−0.160	0.355	0.365	−0.027
PTA	**0.534 (*p* = 0.003)**	–	**0.398 (*p* = 0.049)**	0.314	0.350	0.140	−0.339	0.181	0.406	−0.051	0.074	0.233
Co-located	**0.414 (*p* = 0.028)**	0.059	0.08	0.222	0.006	0.363	0.243	−0.421	−0.49	0.172	0.177	−0.019
Spatially separated	**0.459 (*p* = 0.014)**	**0.419 (*p* = 0.027)**	0.262	0.138	0.329	0.227	−0.258	0.451	0.324	0.32	0.052	−0.528
SRM	0.064	−0.261	−0.123	0.125	−0.254	0.202	**0.459 (*p* = 0.024)**	**−0.735(*p* = 0.004)**	**−0.733 (*p* = 0.004)**	−0.004	0.161	0.300

*Relationships which were found to have p-values of 0.05 or less are indicated in bold with the respective p-value listed below the R-value*.

**Figure 7:**
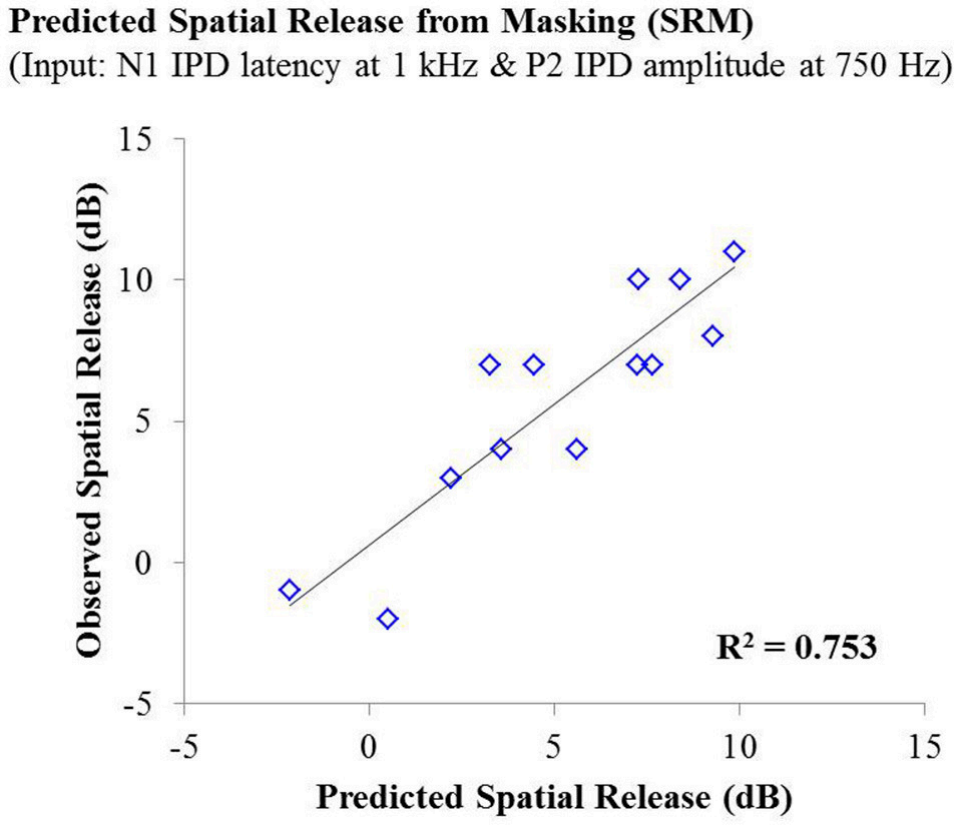
**Observed SRM measurements for individuals in experiment #1 plotted as a function of the amount of spatial release from masking predicted by a linear regression model including the N1 IPD response latency at 1,000 Hz and the P2 IPD response amplitude 750 Hz**. This model yielded an adjusted *R*^2^ value of 0.753.

Lastly, stepwise multiple linear regression was carried out to determine if behavioral performance on either condition the speech test or overall SRM could be predicted by physiological IPD response data, age, or hearing thresholds of participants. Adjusted *R*^2^ values are reported to account for the number of predictors in each model (Miles, 2014). Stepwise linear regression analysis predicting performance on the co-located and the spatially separated condition of the speech task revealed no significant predictive factors, indicating that not age, hearing thresholds, or any IPD indices was sufficient to predict performance on these conditions. However, stepwise linear regression analysis of overall SRM, including the possible predictor variables of age, hearing threshold, and all IPD indices in response to 750 and 1,000 Hz carriers, revealed that more than 75% of the variance in SRM among participants was accounted for by the latency of the N1 peak in response to the 1,000 Hz carrier (standardized β = −0.792) and the amplitude of the P2 peak in response to the 750 Hz carrier (standardized β = 0.507), *F*_(2, 10)_ = 19.265; *p* < 0.001; adjusted *R*^2^ = 0.753. This relationship is shown in Figure 7.

### Experiment #2

**Figure 8:**
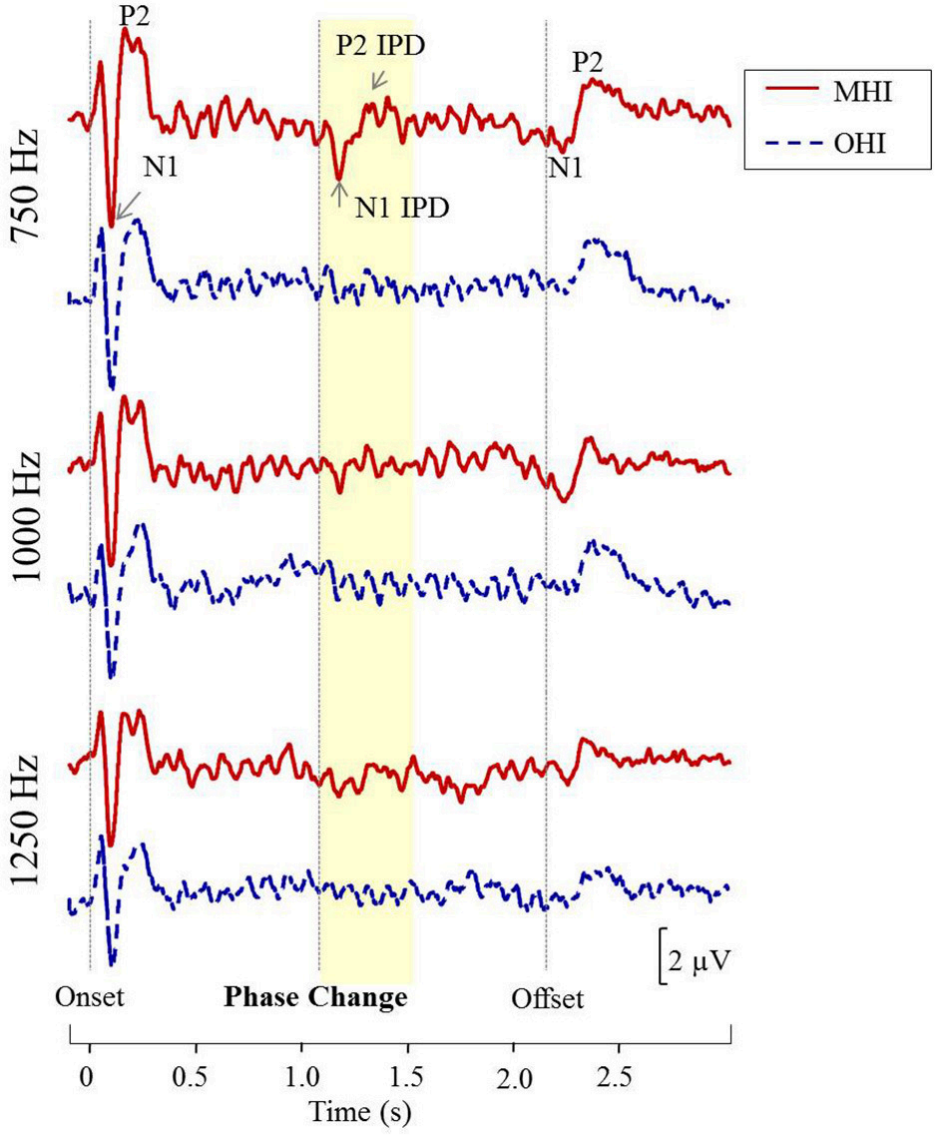
**Grand averaged waveforms for each group in experiment #2**. The yellow segment highlights the portion of the waveforms reflecting response to the phase shift, with respective N1 IPD and P2 IPD peaks labeled.

Figure 8 shows grand averaged waveforms in response to each of the three carrier frequencies, with MHI participant averages shown in red and OHI participant averages shown in dashed blue. Both groups demonstrated clear N1 and P2 onset and offset responses across all carrier frequencies, though the amplitude of the offset response is visibly diminished in response to the 1,250 Hz stimulus compared to responses to either 750 or 1,000 Hz stimuli. The group difference in response to the IPD is apparent in response to 750 Hz stimuli where the MHI listeners clearly show a response while the OHI listeners do not. Compared to their response to 750 Hz stimuli, the grand averaged response of the MHI group to the 1,000 Hz IPD is markedly reduced and is again absent in the OHI group. Neither group shows any discernable response to the IPD at 1,250 Hz.

Table 3 shows the group average N1 and P2 latencies and amplitudes measured from the vertex electrode in response to the onset, IPD, and offset of the 750 and 1,000 Hz stimuli. In response to the 750 and 1,000 Hz stimuli, all individuals in the MHI group had identifiable responses to the stimulus onset and all but one had responses to the stimulus offset, while 11 out of the total 17 participants in this group demonstrated identifiable responses to the IPDs. Responses in the OHI listener group were similar such that all 16 participants had clear onset responses, all but one had identifiable offset responses, and nine individuals had IPD responses when presented with the 750 Hz stimulus. In response to the 1,000 Hz stimulus, all individuals had clear onset responses, but three were lacking clear offset responses, and nine were lacking IPD responses. A one-way ANOVA indicated that MHI listeners had significantly larger N1 IPD response amplitudes compared to OHI listeners when presented with 750 Hz stimuli [*F*_(1, 18)_ = 4.461; *p* = 0.049]. Such a group difference was not found when assessing the onset response to the 750 Hz stimulus [*F*_(1, 31)_ = 0.861; *p* = 0.361] suggesting that the difference found for the IPD response was not due to differences in basic sensitivity to sound energy between the two groups.

**Table 3:** **Group mean (SD) latency and amplitudes of N1 and P2 peaks in response to 750 and 1,000 Hz stimuli amplitude modulated at 10 Hz in experiment #2**.

		**Onset**		**IPD**		**Offset**	
		**Latency (ms)**	**Amp (μV)**	**Latency (ms)**	**Amp (μV)**	**Latency (ms)**	**Amp (μV)**
**N1 PEAKS**							
750 Hz	MHI	104.6 (11.3)	−2.9 (1.4)	1,249.1 (44.6)	−2.7 (1.8)	2,309.2 (17.8)	−2.2 (2.0)
	OHI	107.2 (9.4)	−2.0 (1.6)	1,253.8 (45.8)	−1.1 (1.4)	2,315.6 (19.9)	−0.7 (1.3)
1,000 Hz	MHI	105.4 (11.1)	−2.9 (1.4)	1,262.2 (49.9)	−2.1 (1.5)	2,312.4 (22.3)	−2.0 (1.6)
	OHI	108.1 (13.6)	−2.2 (2.0)	1,305.1 (90.3)	−1.0 (1.0)	2,323.8 (31.4)	−1.2 (1.2)
**P2 PEAKS**							
750 Hz	MHI	187.8 (33.6)	2.7 (2.4)	1,323.7 (36.4)	0.3 (2.1)	2,407.6 (25.4)	1.1 (1.6)
	OHI	213.2 (38.1)	2.1 (1.2)	1,308.5 (35.8)	0.3 (1.0)	2,419.9 (31.9)	1.7 (1.3)
1,000 Hz	MHI	184.9 (32.7)	2.5 (2.1)	1,318.4 (51.3)	0.2 (1.2)	2,408.5 (29.5)	1.2 (1.5)
	OHI	196.6 (39.8)	1.1 (1.7)	1,369.7 (78.1)	0.5 (0.9)	2,419.9 (26.1)	1.0 (0.9)

*Subject groups included middle-age hearing-impaired (MHI) and older hearing-impaired (OHI) participants. Data are shown for responses to stimulus onset, interaural phase shift (IPD) occurring 1,100 ms post-stimulus onset, and stimulus offset occurring at 2,200 ms*.

Individual TMR thresholds measured in both the co-located and the spatially separated conditions of the speech-in-noise task are plotted in Figure 5 (right panel). Overall, the MHI group performed better in the co-located condition with an average TMR threshold of 1.7 dB (SD: 2.9) while the OHI group had an average threshold of 3.3 dB (SD: 1.9 dB), but this difference did not reach statistical significance [*F*_(1, 31)_ = 3.231; *p* = 0.082]. In the spatially separated condition, the MHI group again outperformed the older listeners, achieving an average TMR threshold of −2.8 dB (SD = 3.7 dB) compared to the OHI group's average threshold of 1.0 dB (SD = 3.3 dB). This difference was statistically significant [*F*_(1, 31)_ = 9.716; *p* = 0.004]. Although the difference in SRM between the groups was much larger than in experiment #1, with the MHI group averaging 4.7 dB (SD = 4.2 dB) and the OHI group averaging 2.2 dB (SD = 3.8), the difference between the groups failed to achieve statistical significance [*F*_(1, 14)_ = 1.188; *p* = 0.360] due to the large within-group variability.

**Figure 9:**
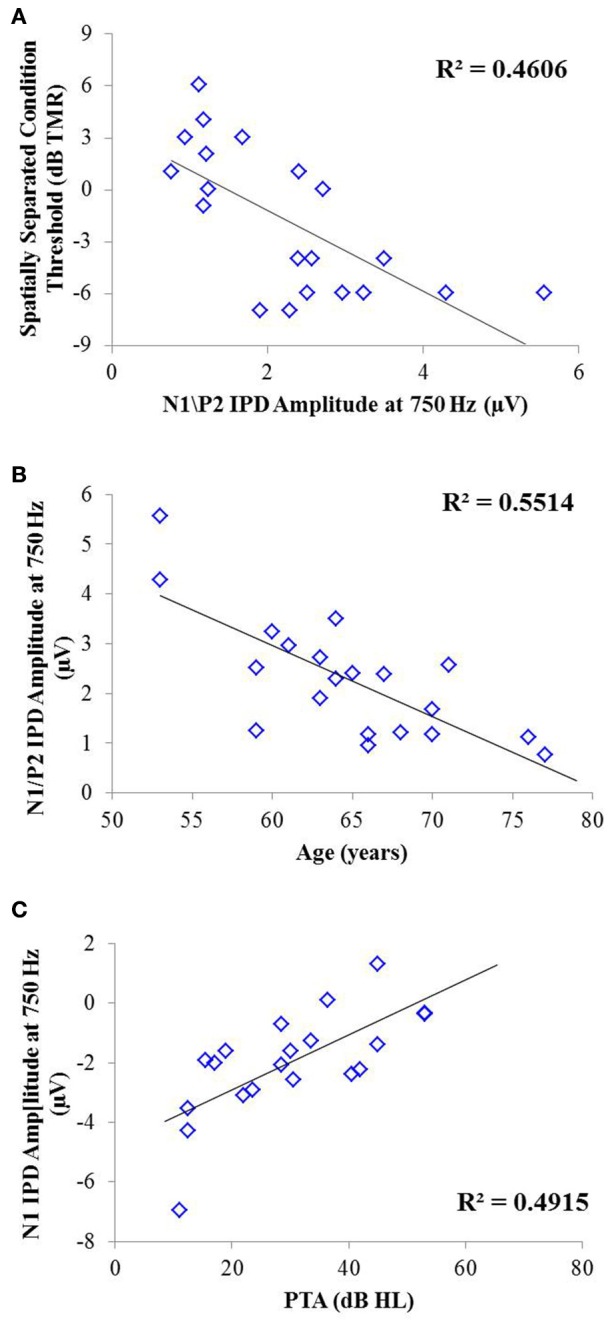
**Significant correlations found between IPD measures and SRM in experiment #2 at the *p* < 0.002 criterion level**. **(A)** Correlation between the spatially separated condition of the speech-in-noise task with the N1/P2 IPD amplitude. **(B)** Correlation between the N1/P2 IPD amplitude and participant age. **(C)** Correlation between the N1 IPD amplitude and participants' pure tone average (PTA) threshold.

Correlation analysis was conducted to assess the strength and significance of relationships between age, average pure tone thresholds, speech recognition in noise, and the latencies and amplitudes of the N1 and P2 components of the IPD in response to 750 Hz stimuli. The results of this analysis are presented in Table 4. As for experiment #1, relationships which reached significance at the *p* < 0.05 criterion level are bolded with the respective *p*-values listed in parentheses below. Application of the Bonferroni correction indicates a significance factor of *p* < 0.002 level, which accounts for the 26 correlations presented for each frequency in experiment #2. Using this strict criterion, the relationship between N1/P2 IPD amplitude and performance in the spatially separated condition remained statistically significant (Figure 9A), as did the relationship between N1/P2 IPD amplitude and age (Figure 9B), and N1 amplitude and PTA (Figure 9C). In addition, relationships between age and PTA, and between PTA and performance on the spatially separated speech task were also significant using the stringent Bonferroni correction. Several other relationships were significant at less stringent criterion levels (Table 4).

**Table 4:** **Correlations (*r* values) between performance on each condition of the speech recognition task and age, pure tone average (PTA) hearing thresholds, and measures of the interaural phase difference (IPD) protocol for participants in experiment #2**.

	**Age**	**PTA**	**IPD Measures: 750 Hz**				
			**N1 Lat**.	**P2 Lat**.	**N1 Amp**.	**P2 Amp**.	**N1/P2 Amp**.
Age	−	**0.560 (*p* < 0.001)**	0.055	−0.347	0.340	−0.189	**−0.742 (*p* < 0.001)**
PTA	**0.560 (*p* < 0.001)**	−	−0.153	−0.081	**0.701 (*p* < 0.001)**	**0.471 (*p* = 0.036)**	−0.359
Co-located	**0.358 (*p* = 0.041)**	0.189	0.163	0.132	−0.032	0.132	−0.336
Spatially separated	**0.473 (*p* = 0.005)**	**0.557 (*p* < 0.001)**	−0.416	**−0.546 (*p* = 0.013)**	**0.510 (*p* = 0.022)**	0.036	**−0.679 (*p* = 0.001)**
SRM	−0.234	**−0.420 (*p* = 0.015)**	**0.517 (*p* = 0.02)**	**0.625 (*p* = 0.003)**	**−0.522 (*p* = 0.018)**	−0.221	**0.443 (*p* = 0.050)**

*Relationships which were found to have p-values of 0.05 or less are indicated in bold with the respective p-value listed below the r-value*.

**Figure 10:**
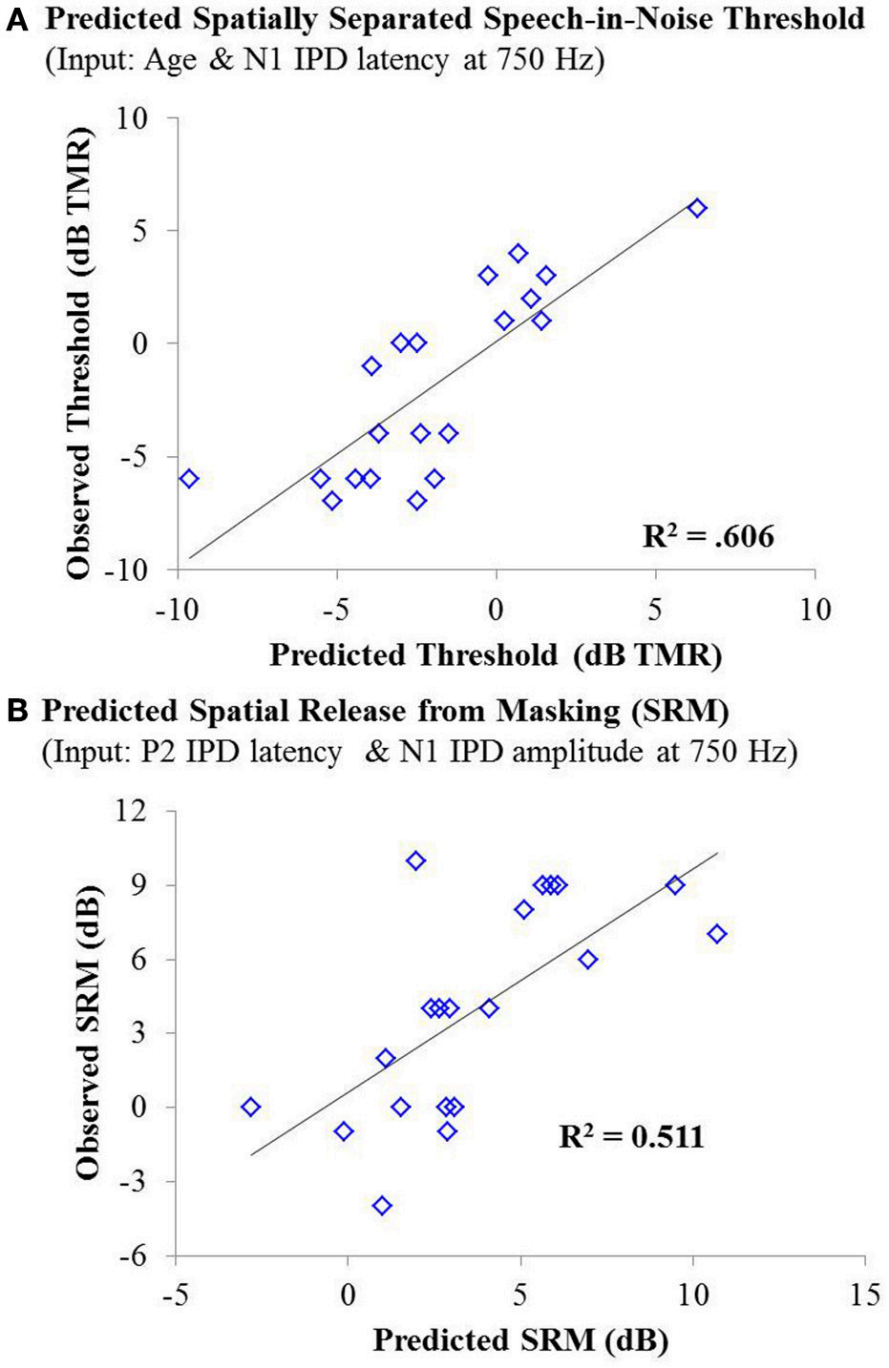
**(A)** Individual thresholds measured in the spatially separated speech-in-noise condition for participants in experiment #2 plotted as a function of thresholds predicted by a linear regression model based on participant age and the latency of the N1 IPD peak in response to 750 Hz stimuli. This model yielded an adjusted *R*^2^ value of 0.606. **(B)** Observed spatial release from masking measurements for individuals in experiment #2 plotted as a function of the amount of spatial release from masking predicted by a linear regression model based on the N1 IPD amplitude and P2 IPD latency in response to the 750 Hz carrier stimulus. This model yielded an adjusted *R*^2^ value of 0.511.

As with experiment #1, multiple linear regression was carried out in order to determine if age, hearing thresholds, or physiological IPD responses were predictive of performance on either condition of the speech recognition task or overall SRM. For the co-located condition of the speech task, stepwise linear regression indicated that participant age provided a small but significant predictive value, accounting for approximately 13% of the variability among participants on this speech test condition, *F*_(1, 42)_ = 7.381; *p* = 0.010; adjusted *R*^2^ = 0.129. For the spatially separated condition of the speech task, the combination of participant age (standardized β = 0.690) and the latency of the N1 IPD peak in response to 750 Hz (standardized β = −0.454) accounted for more than 60% of the variance in performance across participants, *F*_(2, 17)_ = 15.594, *p* < 0.001; adjusted *R*^2^ = 0.606. The relationship between the model predictions and measured thresholds is shown in the top panel of Figure 10. With regard to SRM, stepwise linear regression indicated that the best predictors included the N1 IPD amplitude and P2 IPD latency which together were predicted to account for approximately 51% of the variance among the participants (adjusted *R*^2^ = 0.511). The relationship between the observed and predicted SRM values using this model is displayed in the bottom panel of Figure 10.

## Discussion

The studies presented here investigated the potential for AEP measures to objectively assess the neural encoding of IPD, and explored the relationships between neural encoding of IPD, aging, and hearing loss on the ability to benefit from spatial separation between target and maskers to improve speech recognition performance. Our findings reveal that not only can AEP measures be used as noninvasive physiological markers of IPD, but these measures are significantly predictive of SRM when measured using a speech-in-speech masking task among YNH and MNH listeners (experiment #1). Even amongst MHI and OHI presbyacusic listeners, IPD responses were better predictors of performance in the spatially separated condition and of overall SRM than were either participant age or pure tone hearing thresholds (experiment #2). It is worth noting that the response to the IPD measured here was not a direct measure of binaural processing of IPD, which is thought to occur in the brainstem (Stecker and Gallun, 2012), but rather was a reflection of the response of generators in or near the auditory cortex. This suggests either that the binaural information may have been extracted more poorly in the brainstem or encoded less effectively at the cortical level. The similarity of the response to the onset of the stimulus suggests that the auditory system was processing this change as an indication that a new auditory event had occurred, and that this ability to recognize the onset of the event based on binaural information varied among listeners. In this context, the finding of a strong relationship between IPD responses and the benefits of spatial separation for speech recognition provides compelling confirmation of current models of how the brain performs spatial processing tasks. It is increasingly well known that the ability to utilize binaural temporal cues to improve speech understanding in difficult listening environments varies considerably even among normally hearing individuals (Ruggles et al., 2011; Bharadwaj et al., 2015; Spencer et al., 2016). This variability was reflected in the results of experiment #1 wherein young and middle-age participants with relatively good pure tone thresholds still displayed a range of SRM from −2 to 11 dB. The latency of both N1 and P2 IPD values in response to 1,000 Hz were strongly correlated with SRM performance (Figures 6A,B, respectively), with the N1 IPD latency in response to 1 kHz and the P2 IPD amplitude in response to 750 Hz accounting for more than 75% the variance in SRM for these participants (Figure 7).

Though both aging and hearing loss have been linked to poor performance on behavioral measures requiring sensitivity to binaural cues (Gelfand et al., 1988; Gallun et al., 2013, 2014), neither were significantly correlated with SRM in experiment #1. One possible explanation is that neither age nor hearing loss was great enough to affect SRM in these listeners. Results from experiment #2 suggest that hearing loss may play a larger role than age in determining benefit from SRM as evidenced by the significant correlation between PTA and SRM but not between age and SRM (Table 4). However, similar to the results found in experiment #1, linear regression analysis indicated that the latency of P2 IPD and the amplitude of N1 IPD peaks can account for approximately 51% of the variance in SRM performance in experiment #2, with no contribution of either hearing loss or age (Figure 10). The relationship between IPD measures and SRM among the participants in experiment #2 is striking in part because hearing loss is often cited as the largest contributing factor to speech-in-noise recognition difficulties among older listeners (Humes, 2002, 2007), but the current study found no significant predictive value of either age or hearing loss on SRM. This may reflect the fact that the IPD measure is sensitive to the decreased audibility of the signal arising from cochlear hearing loss as well as being a measure reflecting the neural encoding of temporal cues necessary to achieve SRM, a property that may be degraded with both aging and hearing loss. Regardless, these data indicate that the IPD measurement provides crucial information about spatial speech-in-noise processing which cannot be assessed simply using age and audiometric data.

Both age and hearing loss affect the ability to detect interaural temporal cues, but in differentiable ways. For example, participants in experiment #1 covered a fairly wide age range but had minimal hearing loss. Neither age nor PTA was significantly correlated with SRM in these listeners, though age was weakly correlated with performance in the co-located condition and both age and PTA were weakly correlated with performance in the spatially separated condition. The correlations between age and both conditions of the speech-in-speech task may indicate that even in middle age and without hearing loss, listeners are losing the ability to detect monaural cues such as phase locking to the fine structure of speech. This results in poorer performance in co-located conditions during which listeners must rely upon monaural cues, but also may impact sensitivity to binaural cues, leading to decreased performance in the spatially separated condition. Correlations were found between both age and PTA on performance on the spatially separated task, indicating that even mild hearing loss may compound the effects of aging and thus reduce sensitivity to binaural cues. However, the linear regression model indicates that the effects of aging more heavily impact performance in both speech-in-speech conditions that does hearing loss in these listeners. Similar results were found in experiment #2 such that age was weakly correlated with performance in the co-located condition and both age and PTA were weakly correlated with performance in the spatially separated condition. However, the strength of the correlation between PTA and performance on the spatially separated speech task was stronger than that between age and performance on this condition. Further, linear regression modeling indicated that PTA accounted for a greater portion of the variance in performance on the spatially separated condition than did age in spite of the advanced age of these participants.

This pattern of results implies that while aging has the capacity to negatively affect detection and utilization of both monaural and binaural cues, these effects are essentially swamped by the presence of moderate and greater levels of high-frequency hearing loss common in aging populations. These findings add physiological evidence to a growing body of research regarding the impacts of aging compared to hearing loss on the encoding and use of temporal information (Souza et al., 2007; Gallun et al., 2013, 2014). For example, Hopkins and Moore (2011) reported that behavioral thresholds for detection of temporal fine structure were significantly greater in older listeners compared to young listeners and that these thresholds were unrelated to measures of frequency selectivity. This suggests that the physiological effects that accompany aging impact the ability to detect temporal fine structure independently of broadened frequency tuning curves associated with cochlear hearing loss. Further, King et al. (2014) reported that after accounting for age, pure tone hearing thresholds were correlated with the ability to detect IPD within stimulus temporal fine structure but not envelope, while age was significantly correlated with both detection of IPD in the stimulus fine structure and the envelope.

Robust IPD responses require both synchronous phase locking of the neural response to fine structure of auditory stimuli and precise interaural correlation. Hence, the results of the present study suggest that much of the variance in individual ability to benefit from spatial separation results from differences in encoding of interaural temporal information. The relative importance of low frequency interaural timing differences (ITDs) for spatial processing benefit is in line with previous studies indicating that use of ITD cues dominates sound localization in the horizontal plane, even when all normal spatial cues are available (Macpherson and Middlebrooks, 2002). Further, because objective IPD measures are such strong predictors of spatial processing in normally hearing individuals, the AEP measure of IPD encoding may provide a useful future clinical measure in individuals suspected of having spatial or binaural temporal processing disorders (Glyde et al., 2011; Cameron et al., 2014).

The use of AEPs rather than AEFs to measure neural responses to IPDs holds potential advantages to those exploring binaural processing and speech-in-noise perception in both research and clinical domains. Not only are AEPs much less costly and more widely available than AEF, but AEPs are also sensitive to both tangential and radial current sources while AEF detects only tangential sources. This is a potentially important difference for auditory studies since the auditory cortex is located on Heschl's gyrus within the depths of the Sylvian fissure, and thus activity from this region is best captured using both radial and tangential sources (Picton et al., 1999). The additional information available in AEP measures might account for why we were to acquire reliable IPD recordings from a small number of stimulus presentations compared to the much larger number of stimulus presentations used in previous AEF work (Ross et al., 2007a,b). Otherwise, our results were quite similar to those reported in the previous AEF study of IPD detection across different age groups (Ross et al., 2007a). Ross and colleagues' AEF data revealed an inverse relationship between participant age and the maximum carrier frequency at which a participant could detect the IPD. The results of Experiment #1 in the current study show a similar trend in that our younger participants were more likely to have discernable IPD responses to the 1,000 Hz carrier than their older counterparts (Figure 4), suggesting an age-related decline in the upper frequency limit of physiological IPD detection. This pattern was maintained in experiment #2 in which the majority of participants had IPD responses to the 750 Hz stimulus, but fewer participants had IPD responses when presented with the 1,000 Hz stimulus. In addition, the results of experiment #2 demonstrated that the MHI group had significantly more robust IPD responses at 750 Hz compared to the OHI group (Figure 8). AEF work also has revealed a positive correlation between participant age and the latency of P2 IPD response similar to the effect found in experiment #1 of the current study. However, our current results suggest that even small amounts of hearing loss may reduce IPD sensitivity and that the age group differences in this study as well as previous work may be related more strongly to the hearing loss differences between the groups rather than the age differences.

In summary, current study results reveal insight into the effects of aging and hearing loss on the encoding and use of interaural timing information to improve speech recognition performance in noise, providing an essential bridge between behavioral data and underlying auditory physiology. The AEP paradigm described herein provides a simple, rapid, and widely accessible means of assessing one's ability to encode IPD cues. The results of this AEP paradigm clearly demonstrate that in the young and middle-aged normally hearing listener, simple encoding and comparison of temporal fine structure information between the two ears is adequate to predict a very large proportion of the variance in benefit from spatial separation between a target talker and distracting talkers. Further, in the spatially separated condition of the speech-in-speech recognition task, we found that adding pure tone hearing thresholds along with IPD data into a linear regression model improved the predictive value of the model while the addition of age information did not. This suggests that the IPD protocol captures the aspects of aging relevant to the encoding of interaural timing information and subsequent performance in spatially separated speech recognition tasks, while the influence of hearing loss provides additional contributions to behavioral performance that are not completely accounted for by IPD encoding. Our findings are exciting in that they reveal the potential for AEP measures to further our understanding of spatial processing. However, it is important to remember that this study was exploratory in nature and thus future work is needed to confirm these results. With additional work, the AEP measure of IPD encoding may provide a useful future clinical measure in individuals suspected of having spatial processing disorders, as well as a helpful measure for studying changes in these processes across the lifespan (Glyde et al., 2011; Cameron et al., 2014).

## Ethics statement

Department of Veterans Affairs Portland Healthcare System Institutional Review Board. The informed consent document, approved by the VA Portland IRB, was discussed with all participants after providing ample time for them to review the document on their own. All critical aspects of the informed consent process were discussed including what would happen during the study, their rights as study participants, and how the data would be used during and after study completion. No vulnerable populations were included in the current research.

## Author contributions

MP: Data acquisition and analysis, data interpretation, drafting of initial document and revisions, and approval of final version. RF: Conception and design of work, interpretation of data, critical review of manuscript for important intellectual content, and approval of final version. FG: Conception and design of work, interpretation of data, critical review of manuscript for important intellectual content, and approval of final version. All authors (MP, RF, and FG) agree to be accountable for all aspects of the work, ensuring that questions related to the accuracy or integrity of any part of the work are appropriately investigated and resolved.

### Conflict of interest statement

The authors declare that the research was conducted in the absence of any commercial or financial relationships that could be construed as a potential conflict of interest.
